# Control of total GFP expression by alterations to the 3′ region nucleotide sequence

**DOI:** 10.1186/1475-2859-12-68

**Published:** 2013-07-08

**Authors:** Sang Jun Lee, Eun Hee Park, Young Ok Kim, Bo Hye Nam, Dong Gyun Kim

**Affiliations:** 1 Biotechnology Research Division, National Fisheries Research & Development Institute, Busan 619-902, Korea

**Keywords:** Hydrophilic C-terminal end, Longer 5′ poly(A) coding sequence, mRNA tertiary structure, Synonymous codon substitution in the 3′ region, Positional recovery effect, Inserted trinucleotides in the 3′-UTR, Translational and transcriptional regulation, Transcriptional termination

## Abstract

**Background:**

Previously, we distinguished the *Escherichia coli* type II cytoplasmic membrane translocation pathways of Tat, Yid, and Sec for unfolded and folded soluble target proteins. The translocation of folded protein to the periplasm for soluble expression via the Tat pathway was controlled by an N-terminal hydrophilic leader sequence. In this study, we investigated the effect of the hydrophilic C-terminal end and its nucleotide sequence on total and soluble protein expression.

**Results:**

The native hydrophilic C-terminal end of GFP was obtained by deleting the C-terminal peptide LeuGlu-6×His, derived from pET22b(+). The corresponding clones induced total and soluble GFP expression that was either slightly increased or dramatically reduced, apparently through reconstruction of the nucleotide sequence around the stop codon in the 3′ region. In the expression-induced clones, the hydrophilic C-terminus showed increased Tat pathway specificity for soluble expression. However, in the expression-reduced clone, after analyzing the role of the 5′ poly(A) coding sequence with a substituted synonymous codon, we proved that the longer 5′ poly(A) coding sequence interacted with the reconstructed 3′ region nucleotide sequence to create a new mRNA tertiary structure between the 5′ and 3′ regions, which resulted in reduced total GFP expression. Further, to recover the reduced expression by changing the 3′ nucleotide sequence, after replacing selected C-terminal 5′ codons and the stop codon in the ORF with synonymous codons, total GFP expression in most of the clones was recovered to the undeleted control level. The insertion of trinucleotides after the stop codon in the 3′-UTR recovered or reduced total GFP expression. RT-PCR revealed that the level of total protein expression was controlled by changes in translational or transcriptional regulation, which were induced or reduced by the substitution or insertion of 3′ region nucleotides.

**Conclusions:**

We found that the hydrophilic C-terminal end of GFP increased Tat pathway specificity and that the 3′ nucleotide sequence played an important role in total protein expression through translational and transcriptional regulation. These findings may be useful for efficiently producing recombinant proteins as well as for potentially controlling the expression level of specific genes in the body for therapeutic purposes.

## Background

Previously, we characterized the N-terminal specific *Escherichia coli* type II cytoplasmic membrane translocation pathways of Tat, Yid, and Sec for periplasmic soluble expression of unfolded and folded target proteins [1]. Using green fluorescent protein (GFP) with short N-terminal polypeptides exhibiting an isoelectric point (pI) and hydrophilicity separately, with an anchor sequence, M(X)(Y)[pI]-anchor-8×Arg(hydrophilicity)-GFP [pI and hydrophilicity separately], the bulky, folded protein was able to pass efficiently through the Tat pathway via the translocon with the largest diameter; moreover, passage was controlled by the pI value of the N-terminus in the order of acidic > neutral. However, for GFP carrying a short N-terminal string of hydrophilic amino acids followed by Met without an anchor sequence; i.e., Met-hydrophilic sequence(6×Glu, 6×Lys, etc.)-GFP [pI and hydrophilicity together], translocation of the folded protein to the periplasm for soluble expression through the Tat pathway was controlled by the hydrophilic leader sequence (acidic and alkaline).

However, in *E. coli*, there are a further two types of soluble recombinant protein expression techniques using the C-terminal tags, which are distinct from the above N-terminal specific type II cytoplasmic membrane translocation pathways. Firstly, another genetically defined type I secretory mechanism has been used for export of target proteins to the culture medium. Although several type I transporters can been used for recombinant protein production, the *E. coli* α-hemolysin (HlyA) transporter is by far the most popular. The C-terminal region of HlyA contains all the information required for efficient translocation and can therefore be used as a signal sequence for recombinant protein targeting [2]. Secondly, C-terminal extensions have been used for soluble target protein expression in the cytoplasm [3]. The cytoplasimic solubility of the C-terminal extensions are presumably close to the above cytoplasmic membrane translocation pathways of the type II secretory mechanism; however, the exact pathway and its specificity have not yet been defined.

In this study, based on the confirmed result that the hydrophilic N-terminus attached to GFP had the Tat pathway specificity shown previously [1], we aimed to identify the hydrophilic role of the native C-terminal end of GFP (MDELYK; 6 aa; hydrophilicity [hy], +0.35) for soluble expression through the Tat pathway. We constructed the corresponding clones after deleting the LeuGlu(LE)-6×His (6H; 6 aa; hy, –0.28) peptide, derived from pET22b(+), and evaluated the total and soluble GFP expression levels. Our results show that two of three deleted clones with the hydrophilic C-terminal end induced slightly higher levels of total and soluble GFP expression than the parental clones, caused by increased Tat pathway specificity. Therefore, we confirmed that the hydrophilic C-terminal could enhance the solubility of the folded GFP expression through the established Tat pathway of the type II secretory mechanism. This suggests that the hydrophilic C-terminals, or any hydrophilic C-terminal extensions that enhance the cytoplasmic solubility of the folded target proteins, belong to the Tat translocon of the type II cytoplasmic membrane translocation pathways.

However, the third deleted clone with a hydrophilic C-terminal end exhibited dramatically reduced total and soluble GFP expression. Therefore, we concluded that the reduction in total and soluble GFP expression in the one deleted clone was related to reconstruction of the nucleotide sequence around the stop codon in the 3′ region, but not related to the general property of the native hydrophilic C-terminal end of GFP. We proved that the longer 5′ poly(A) coding sequence interacted with the reconstructed 3′ region sequence to create a new mRNA tertiary structure that caused reduced total GFP expression. Further, to confirm the role of the 3′ region reconstructed sequence, we changed the nucleotide sequence of the 3′ region in the clone, investigated the mRNA and total GFP expression levels, and concluded that the ribonucleotide sequence of the 3′ region plays an important role in the translational and transcriptional regulation of total GFP expression.

## Results and discussion

### Effect of the reconstructed nucleotide sequence around the stop codon on total and soluble GFP expression

In a previous study, highly expressed recombinant GFP with the hydrophilic N-termini ME_6_ (hy, +1.82) and MK_6_ (hy, +1.82), referred to as ME_6_-GFP and MK_6_-GFP, respectively [1], had an “artificial” C-terminal peptide, LeuGlu (the corresponding peptide for the *Xho*I restriction site; referred to as “LE” below)-6×His (His tag; referred to as “6H” below), not specified in the previous study [1], derived from the expression vector pET22b(+) (Additional file 1: Table S3, clones 3 and 5). Thus, we constructed clones to remove the corresponding nucleotide sequence of LE-6H (6H; 6 aa; hy, –0.28), including from the control clone GFP-LE-6H-Stop(TGA) (Additional file 1: Table S3, clone 1), but leaving the native hydrophilic C-terminal end sequence of GFP (MDELYK; 6 aa; hy, +0.35) and native stop codon (TAA). The resulting clones were referred to as GFP-Stop(TAA)-*#* (# represents the deleted, non-coding nucleotide sequence of “*XhoI-6×His*”), ME_6_-GFP-Stop(TAA)-#, and MK_6_-GFP-Stop(TAA)-# (Additional file 1: Table S3, clones 2, 4, and 6).

**Figure 1:**
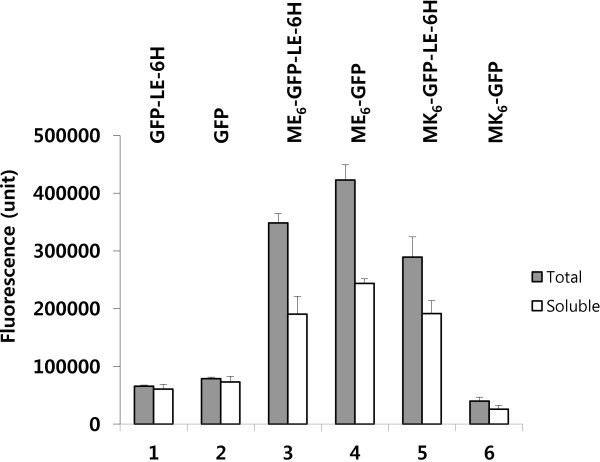
**Effect of the native hydrophilic C-terminus of GFP on total and soluble protein expression.** To obtain GFP expression vectors carrying the native hydrophilic C-terminal sequence of GFP (MDELYK; 6 aa; hy, +0.35) with a native stop codon (TAA), we constructed the C-terminal peptide LeuGlu(LE)-6×His (6H; 6 aa; hy, –0.28)-deleted clones of GFP-Stop(TAA)-# (where # = *XhoI-6*×*His*, the deleted non-coding nucleotide sequence of LeuGlu [*Xho*I restriction site]-6×His and is indicated by italics beyond the stop codon), ME_6_-GFP-Stop(TAA)-#, and MK_6_-GFP-Stop(TAA)-# from the control clones of GFP-LE-6H-Stop(TGA), ME_6_-GFP-LE-6H-Stop(TGA), and MK_6_-GFP-LE-6H-Stop(TGA), respectively, as described in the Methods. The coding region of the *Xho*I restriction site (CTCGAG; LeuGlu)-6×His(CAC) tag, derived from pET22b(+), is referred to as LeuGlu-6×His (LE-6H), and the non-coding region of the same sequence beyond the stop codon is #. Approximately 50 μg of total protein and a counter volume of the soluble fraction were used for fluorescence measurements as described in the Methods. All cultures were performed in triplicate, and results represent the mean value of at least duplicate experiments. Error bars indicate standard deviations. Lanes: 1, GFP-LE-6H-Stop(TGA), control; 2, GFP-Stop(TAA)- #; 3, ME_6_-GFP-LE-6H-Stop(TGA), control; 4, ME_6_-GFP-Stop(TAA)- #; 5, MK_6_-GFP-LE-6H-Stop(TGA), control; and 6, MK_6_-GFP-Stop(TAA)- #.

We investigated the total and soluble GFP expression levels of the LE-6H peptide-deleted (henceforth referred to as simply “deleted”) clones. The total and soluble expression levels of the GFP and ME_6_-GFP proteins from the deleted clones GFP-Stop(TAA)-# and ME_6_-GFP-Stop(TAA)-# were increased to 20.0 and 19.9%, and 17.6 and 27.7%, respectively, compared to those of the undeleted controls (Figure 1, lanes 2 and 4). These results indicate that when the C-terminal end is more hydrophilic, specificity for the largest translocon (i.e., the Tat channel) was increased compared to that of the hydrophilic N-terminus [1]. The increased total protein expression level suggests that the increased Tat pathway specificity helped to synthesize protein in the cytoplasm by a type of non-feedback regulation, secreting the synthesized protein quickly into the periplasm. However, the subsequent soluble protein expression level was generally reflected by the total protein expression level; thus, the primary total protein expression level could be used as an indicator of the soluble protein expression level in this study.

The total and soluble expression levels of MK_6_-GFP from the deleted clone, MK(AAA)_6_-GFP-Stop(TAA)-#, were markedly reduced (Figure 1, lane 6). We concluded that this was caused by reconstruction of the nucleotide sequence around the stop codon in the 3′ region due to deletion of the corresponding nucleotide sequence of the LE-6H peptide. When compared to the other deleted clone, the total and soluble expression of ME_6_-GFP was not reduced in the clone ME(GAA)_6_-GFP-Stop(TAA)-#, which indicates that the 5′ 6×GAA sequence in the clone ME(GAA)_6_-GFP-Stop(TAA)-# was not responsible for the reduction in GFP expression. Therefore, we concluded that the alternative 5′ 6×AAA sequence in the clone MK(AAA)_6_-GFP-Stop(TAA)-# was most likely involved in the dramatic reduction in both total and soluble protein expression.

### Effects of the altered 5′ 6×AAA sequence with an AAG codon on total MK_6_-GFP expression

**Figure 2:**
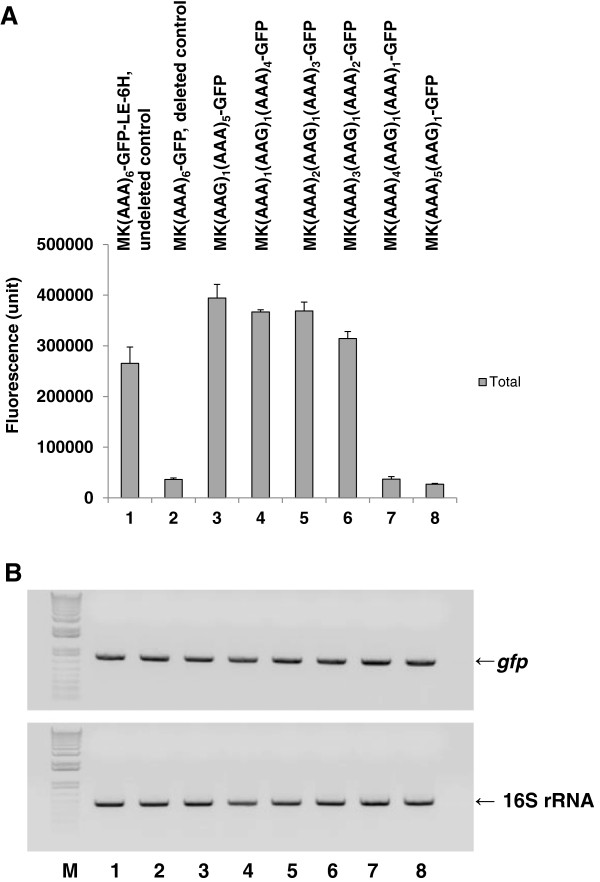
**Comparison of fluorescence (A) and RT-PCR (B) data for various MK**_**6**_**-GFP clones with different orders of 5×AAA and 1×AAG in the K**_**6**_**.** To obtain the corresponding clones, we constructed MK_6_-GFP-Stop(TAA)-# clones with varying sequences of 5×AAA and 1×AAG codons within the K_6_ sequence, as described in the Methods. The determination of total protein fluorescence was conducted as in Figure 1. A semi-quantitative RT-PCR analysis of 1-h IPTG-induced cultures of the MK_6_-GFP clones was conducted at 30°C after 30 cycles, as described in the Methods. To check for saturation of the PCR products before 30 cycles, an RT-PCR analysis was conducted after 20 cycles, but the general thin and unsaturated density band patterns of the 1-h induced cultures were similar amongst the clones (data not shown). The upper and lower bands correspond to the *gfp* and 16S rRNA genes, respectively. We used an Invitrogen 1 kb plus ladder as the DNA size marker. All cultures, mean values, and error bars are as in Figure 1. Lanes: 1, MK(AAA)_6_-GFP-LE-6H-Stop(TGA), undeleted control; 2, MK(AAA)_6_-GFP-Stop(TAA)-#, deleted control; 3, MK(AAG)_1_(AAA)_5_-GFP-Stop(TAA)-#; 4, MK(AAA)_1_(AAG)_1_(AAA)_4_-GFP-Stop(TAA)-#; 5, MK(AAA)_2_(AAG)_1_(AAA)_3_-GFP-Stop(TAA)-#; 6, MK(AAA)_3_ (AAG)_1_(AAA)_2_-GFP-Stop(TAA)-#; 7, MK(AAA)_4_(AAG)_1_(AAA)_1_-GFP-Stop(TAA)-#; and 8, MK(AAA)_5_(AAG)_1_-GFP-Stop(TAA)-#.

To confirm the above hypothesis of the role of the 5′ 6×AAA sequence in the reduction of GFP expression, we replaced the AAA codon (6×AAA = AAA_6_) with a synonymous codon (AAG), designed the order of sequences based on the 5×AAA and 1×AAG codons that comprised the derivative clones from the deleted control clone MK(AAA)_6_-GFP-Stop(TAA)-#, made the corresponding clones (Additional file 1: Table S3, clones 7–12), and tested for total MK_6_-GFP expression. Our results show that the corresponding clones (AAG)_1_(AAA)_5_, (AAA)_1_(AAG)_1_(AAA)_4_, (AAA)_2_(AAG)_1_(AAA)_3_, and (AAA)_3_(AAG)_1_(AAA)_2_ did not reduce total MK_6_-GFP expression (Figure 2A, lanes 3–6), but that the corresponding clones (AAA)_4_(AAG)_1_(AAA)_1_ and (AAA)_5_(AAG)_1_ reduced total MK_6_-GFP expression to a level similar to that of the corresponding deleted control clone, (AAA)_6_ (Figure 2A, lanes 7 and 8). However, the mRNA levels, as determined by semi-quantitative RT-PCR, were similar among all of the clones with a substituted AAG codon (Figure 2B, lanes 3–8). Therefore, we concluded that the reduction in total MK_6_-GFP expression caused by the deleted control clone, MK(AAA)_6_-GFP-Stop(TAA)-#, and its derivative clones, (AAA)_4_(AAG)_1_(AAA)_1_ and (AAA)_5_(AAG)_1_, was affected by translational regulation and not transcriptional regulation (Figure 2, lanes 2, 7, and 8).

In the expression-reduced clone, we first demonstrated the presence of the reciprocal interaction of the longer 5′ poly(A) coding sequences of (AAA)_4_AA-(−:G) and (AAA)_5_AA-(−:G) and the control sequence of (AAA)_6_A--(−−:TG) with the reconstructed ribonucleotide sequence around the stop codon in the deleted control clone, MK(AAA)_6_-GFP-Stop(TAA)-#, and its derivative clones with (AAA)_4_AA- and (AAA)_5_AA- (Figure 2A, lanes 2, 7, and 8); however, the same control sequence, (AAA)_6_A--, would not interact with the authentic (LE-6H-containing) ribonucleotide sequence around the stop codon to reduce protein expression in the undeleted control clone, MK(AAA)_6_-GFP-LE-6H-Stop(TGA) (Figure 2A, lane 1). These results indicate that a new mRNA tertiary structure should be created between the 5′ and 3′ region sequences in the mRNA generated from the deleted control clone MK(AAA)_6_-GFP-Stop(TAA)-# and its derivative clones containing the longer 5′ poly(A) coding sequences of (AAA)_4_AA- and (AAA)_5_AA-, and that this new tertiary structure caused the reduction in total MK_6_-GFP expression.

To distinguish the roles of the 5′ poly(A) coding sequence and its synonymous derivatives in the MK_6_-GFP expression, we investigated the folding energy of the mRNA secondary structure [4,5]; however, the predicted folding energy of the 5′ mRNA sequences from the MK(AAA)_6_ clone and its related clones, substituted with a synonymous codon of K(AAG) in the K_6_ with different total expression levels, did not correlated with the local and entire sequence (data not shown). Particularly, the uncorrelated 5′ mRNA folding energy of the synthetic leader sequences of MK_6_ regions (nt positions −4 to +36) of the MK_6_-GFP clones was not proportional to the mRNA folding energy of the codon start region (nt −4 through +37) of the synonymously mutated constructs for unaltered GFP expression, which was strongly correlated with fluorescence [6]. We believed that one possible reason for the difference in the folding energy of the start codon regions between the two MK_6_-GFP and GFP clones is because the mRNA structures of the artificially attached synthetic MK_6_ leader sequence regions of MK_6_-GFP clones in this study have a highly repeated poly(A) sequence structure at the 5′ region, which might interfere with correct calculation of folding energy.

Furthermore, the MK(AAA)_6_ clone and its derivative clones, substituted with a synonymous codon of K(AAG) in the K_6_, did not show consistent total protein expression levels as calculated by the codon adaptation index (CAI) [7] of the local and entire sequences (data not shown). Therefore, we concluded that the reduction in total MK_6_-GFP expression by the deleted control clone, MK(AAA)_6_-GFP-Stop(TAA)-#, and its derivative clones, containing the longer 5′ poly(A) coding sequences of (AAA)_4_AA- and (AAA)_5_AA-, was not dictated by the folding energy of the mRNA secondary structure or codon bias based on the CAI, but depended primarily on a new mRNA tertiary structure created between the longer 5′ poly(A) coding sequence and the reconstructed 3′ region nucleotide sequence (see below).

### Effect of substituting the C-terminal 5′ codons and stop codon in the 3′ region of the open reading frame (ORF) with synonymous codons on total MK_6_-GFP expression

We recognized that the primary reason for the reduction in total MK_6_-GFP expression by the C-terminal LE-6H peptide-deleted control clone, MK(AAA)_6_-GFP-Stop(TAA)-#, was the reconstructed nucleotide sequence around the stop codon (Figure 1, lane 6). The deleted control clone, MK(AAA)_6_-GFP-TAA-#, had a reconstructed nucleotide sequence in the 3′ region that contained the native hydrophilic C-terminal end of GFP (MDELYK; 6 aa; hy, +0.35) instead of the undeleted LE-6H (6H; 6 aa; hy, –0.28) peptide sequence, the native GFP stop codon (TAA) instead of TGA, and the # sequence (non-coding nucleotide sequence of *XhoI-6×His*) in the 3′-untranslated region (UTR) around the stop codon, as described above. The C-terminal 5′ codons and the stop codon (TAA) were located in the ORF. Therefore, to confirm the role of the 3′ region reconstructed nucleotide sequence in the ORF, we hypothesized that any nucleotide change in the C-terminal 5′ codons and stop codon would affect MK_6_-GFP expression.

**Figure 3:**
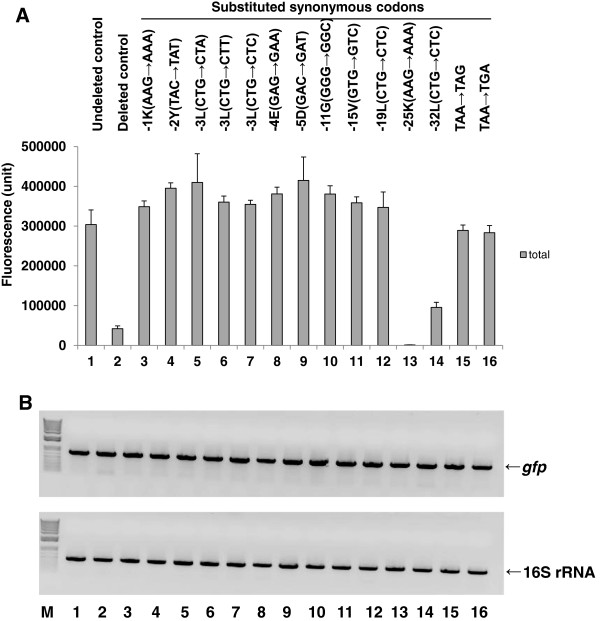
**Comparison of fluorescence (A) and RT-PCR (B) data for MK**_**6**_**-GFP-Stop(TAA)-# clones substituted with synonymous codons, their altered nucleotide positions, clone abbreviations represented by the amino acid position and codons (bold letters).** To obtain the corresponding clones, we replaced the selected C-terminal 5′ codon (positions −32 to −1) and the stop codon within the ORF with a synonymous codon in the MK_6_-GFP-Stop(TAA)-# clone, as described in the Methods. Determinations of the total protein fluorescence and semi-quantitative RT-PCR were conducted as in Figure 2. The data after 30 cycles of RT-PCR were better than those after 20 cycles (data not shown) when comparing the general band density of clones. The upper and lower bands and the size marker are indicated as in Figure 2. All cultures, mean values, and error bars are as in Figure 1. Lanes: 1, MK(AAA)_6_-GFP-LE-6H-Stop(TGA), **undeleted control**; 2, MK(AAA)_6_-GFP-Stop(TAA)-#*,***deleted control**; 3, MK_6_-GFP[C-terminal −1 K(AAG→AAA, 741)]-Stop(TAA)-#, **-1 K(AAG→AAA)**; 4, MK_6_-GFP[C-terminal −2 Y(TAC→TAT, 738)] -Stop(TAA)-#, **-2 Y(TAC→TAT)**; 5, MK_6_-GFP[C-terminal −3 L(CTG→CTA, 735)]-Stop(TAA)-#, **-3 L(CTG→CTA)**; 6, MK_6_-GFP[C-terminal −3 L(CTG→CTT, 735)]-Stop(TAA)-#, **-3 L(CTG→CTT**); 7, MK_6_-GFP[C-terminal −3 L(CTG→CTC, 735)]-Stop(TAA)-#, **-3 L(CTG→CTC)**; 8, MK_6_-GFP[C-terminal −4 E(GAG→GAA, 732)]-Stop(TAA)-#, **-4 E(GAG→GAA)**; 9, MK_6_-GFP[C-terminal −5 D(GAC→GAT, 729)]-Stop(TAA)-#, **-5 D(GAC→GAT)**; 10, MK_6_-GFP[C-terminal −11 G(GGG→GGC, 711)]-Stop(TAA)*-*#, **-11 G(GGG→GGC)**; 11, MK_6_-GFP[C-terminal −15 V(GTG→GTC, 699)]-Stop(TAA)*-*#, **-15 V(GTG→GTC)**; 12, MK_6_-GFP[C-terminal −19 L(CTG→CTC, 687)]-Stop(TAA)*-*#, **-19 L(CTG→CTC)**; 13, MK_6_-GFP[C-terminal −25 K(AAG→AAA, 669)]- Stop(TAA)*-*#, **-25 K(AAG→AAA)**; 14, MK_6_-GFP[C-terminal −32 L(CTG→CTC, 648)]- Stop(TAA)*-*#, **-32 L(CTG→CTC)**; 15, MK_6_-GFP-Stop(TAA→TAG, 744)-#, **TAA→TAG**; and 16, MK_6_-GFP-Stop(TAA→TGA, 743)-#, **TAA→TGA**.

We focused on the effect of single nucleotide changes in the C-terminal 5′ codons and stop codon in the 3′ region of the ORF on total MK_6_-GFP expression. We substituted selected codons amongst the extended C-terminal 5′ codons (positions −32 to −1) and the stop codon with synonymous codons to assess the expression of the unchanged protein coding sequence of MK_6_-GFP. Thus, we replaced the C-terminal 5′ codons of L_−32_------K_−25_-----L_−19_---V_−15_---G_−11_-----D_−5_E_−4_L_−3_Y_−2_K_−1_ and the stop codon with synonymous codons in the deleted control clone, MK(AAA)_6_-GFP-TAA-# (Additional file 1: Table S3, clones 13–26). All clones in which a single codon was replaced with a synonymous codon exhibited marked recovery of total protein expression (Figure 3A, lanes 3–12, 15, and 16), with the exception of those clones with substitutions at L_−32_ (−32 L) and K_−25_ (−25 K), which were located relatively far from the stop codon (Figure 3A, lanes 13 and 14).

Next, we analyzed mRNA expression by semi-quantitative RT-PCR to determine the relationship between total protein and mRNA expression levels over time in the codon-substituted MK(AAA)_6_-GFP-Stop(TAA)-# clone derivatives (Figure 3B). All high-level total protein-expressing clones containing codon replacements at the C-terminus (C-terminal 5′ codon positions −19 to −1) and in the stop codon within the ORF had high levels of mRNA (Figure 3B, lanes 3–12, 15, and 16). In contrast, those clones with synonymous codon substitutions at C-terminal 5′ codon positions −32 and −25 expressed much lower or slightly higher total protein levels, respectively, than that of the deleted control clone, which also exhibited high levels of mRNA (Figure 3B, lanes 13 and 14).

From these results, we recognized that a change in one nucleotide in each of the tested C-terminal 5′ codons (positions −19, -15, -11, -5, -4, -3 [three substitutions], -2, and -1) and the stop codon (two substitutions) substituted in the clone MK(AAA)_6_-GFP-Stop(TAA)-# resulted in recovery of total MK_6_-GFP expression to a level higher or comparable to that of the undeleted control clone, MK(AAA)_6_-GFP-LE-6H-Stop(TGA) (Figure 3A, lanes 3–12, 15, and 16). The substituted nucleotides within the synonymous codons (e.g., G→A, G→T, G→C, C→T, or A→G) in the C-terminal 5′ codon positions −19 to −1 and in the stop codon could overcome the low-level expression of total MK_6_-GFP, but the same was not the case at C-terminal 5′ codon positions −32 and −25 (e.g., G→C, G→A). These results suggested the existence of a positional effect in the 3′ region of mRNA structure, in which changes in distantly located codons (e.g., positions −32 and −25) do not lead to full recovery of the total MK_6_-GFP expression levels.

In previous study [6], statistical analysis showed that the corresponding 3′ region nucleotide positions of the synonymously mutated constructs for unaltered GFP expression were not consistent in gene expression, which represented that total protein expression levels depended upon the substituted nucleotide position with a randomized variation in the whole scale of the significance. However, our plotted results showed that the synonymously changed single nucleotide sequence in the C-terminal 5′-codons (positions −19 to 1) and the stop codon of the 3′ region (nt positions 687 to 744) consistently recovered total MK_6_-GFP expression levels (Additional file 1: Figure S1), which suggested that all of the clones contain an mRNA structure different from the mRNA tertiary structure of the deleted control clone for enhanced translation. Therefore, it seems like that the authentic specified 3′ region (nt positions 687 to 744) plays an important role in creating an mRNA tertiary structure with the longer 5′ poly(A) coding sequence. However, all synonymous codon substitutions showed no co-relationship with total protein expression levels in either the folding energy of the mRNA secondary structure [4,5] in the local and entire sequences (data not shown), or codon bias based on the CAI [7] of the local and global sequence (data not shown), which is similar to the previously reported CAI [6].

Our RT-PCR results showed that all of the clones with synonymous codon substitutions in their C-terminal 5′ codons and stop codon that expressed high or low levels of total protein also had relatively high or comparable mRNA levels (Figure 3A and B), which was unexpected. This result revealed a lack of correlation between a high mRNA level and low total protein expression level, indicating that there was no defect in mRNA synthesis mediated by transcriptional regulation. Thus, we suggest that any single ribonucleotide that is changed within the proper distance of the C-terminal 5′ codons (positions −19 to −1) and stop codon with a synonymous codon could result in a severe deviation from the initial mRNA tertiary structure generated from the deleted, parental control clone, which could subsequently affect the recovery of total MK_6_-GFP expression through recovered translational regulation.

Therefore, it can be concluded that the C-terminal 5′ codons (positions −19 to −1) and stop codon of the *gfp* gene sequence in the 3′ region are involved in generating the mRNA tertiary structure with the longer 5′ poly(A) coding sequence. Any single nucleotide change in the specified 3′ region (nt positions 687 to 744) showed the presence of a positional recovery effect due to the changed mRNA structure from that of the deleted control clone for translational recovery. This structure has a sensitive regulatory mechanism, in which the minimally changed nucleotide sequences in the 3′ region of the ORF could affect the fate of the mRNA tertiary structure, which could induce increased or decreased translational efficiency, resulting in higher or lower total protein expression. This result showed that the internal mRNA tertiary structure is one of the most important factors in translational regulation.

### Effect of inserted trinucleotides beyond the stop codon in the 3′-UTR on total MK_6_-GFP expression

The C-terminal LE-6H peptide-deleted control clone, MK(AAA)_6_-GFP-Stop(TAA)-# (Figure 1, lane 6), contains a reconstructed nucleotide sequence in the 3′ region, which harbors the native hydrophilic C-terminal end of GFP (MDELYK; 6 aa; hy, +0.35), the replaced native GFP stop codon (TAA), and the relocated # sequence (the non-coding nucleotide sequence of *XhoI-6×His*) in the 3′-UTR around the stop codon, as described above. The non-coding nucleotide sequence of # is located just beyond the stop codon in the 3′-UTR. To confirm the role of the 3′-UTR in the reconstructed 3′ region nucleotide sequence, we hypothesized that any inserted nucleotide beyond the stop codon in the 3′-UTR would affect MK_6_-GFP expression.

**Figure 4:**
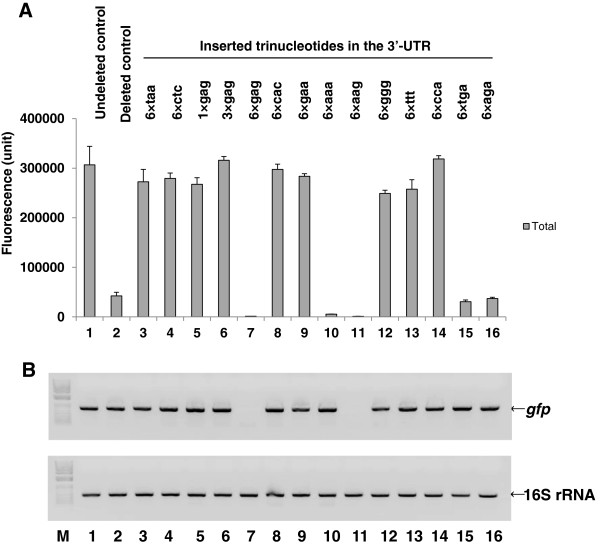
**Comparison of the fluorescence (A) and RT-PCR (B) data from the MK**_**6**_**-GFP-Stop(TAA)-(xxx)-# clones and their abbreviations represented by the inserted trinucleotide (in bold type).** To obtain the corresponding clones, we inserted single or repeated trinucleotides just beyond the stop codon in the 3′-UTR, outside of the ORF, in the MK_6_-GFP-Stop(TAA)-(xxx [= the inserted trinucleotide, indicated in lowercase letters])-# clone, as described in the Methods. Determinations of the total protein fluorescence and semi-quantitative RT-PCR were conducted as in Figure 2. Data from products generated after 30 cycles of RT-PCR from the 1-h induced cultures were compared among the clones. The upper and lower bands and the size marker are indicated as in Figure 2. All cultures, mean values, and error bars are as in Figure 1. Lanes: 1, MK_6_-GFP-LE-6H-Stop(TGA), **undeleted control**; 2, MK_6_-GFP-Stop(TAA)-#, **deleted control**; 3, MK_6_-GFP-Stop(TAA)-6×taa-#, **6× taa**; 4, MK_6_-GFP-Stop(TAA)-6×ctc-#, **6× ctc**; 5, MK_6_-GFP-Stop(TAA)-1×gag-#, **1× gag**; 6, MK_6_-GFP-Stop(TAA)-3×gag-#, **3× gag**; 7, MK_6_-GFP-Stop(TAA)-6×gag*-*#, **6× gag**; 8, MK_6_-GFP-Stop(TAA)-6×cac-#, **6× cac**; 9, MK_6_-GFP-Stop(TAA)-6×gaa-#, **6× gaa**; 10, MK_6_-GFP-Stop(TAA)-6×aaa-#, **6× aaa**; 11, MK_6_-GFP-Stop(TAA)-6×aag-#, **6× aag**; 12, MK_6_-GFP-Stop(TAA)-6×ggg-#, **6× ggg**; 13, MK_6_-GFP-Stop(TAA)-6×ttt-#, **6× ttt**; 14, MK_6_-GFP-Stop(TAA)-6×cca-#, **6× cca**; 15, MK_6_-GFP-Stop(TAA)-6×tga-#, **6× tga**; and 16, MK_6_-GFP-Stop(TAA)-6×aga-#, **6× aga**.

We analyzed the reconstructed 3′ nucleotide sequence, taa ctc gag cac cac cac cac cac cac, in the TAA(stop codon)-# (*ctc gag[XhoI]-6*×*cac[His]*) region of the deleted control clone, MK(AAA)_6_-GFP-Stop(TAA)-# (Figure 1, lane 6) and assessed the four different kinds of basic component trinucleotides (taa, ctc, gag, and cac). Next, MK(AAA)_6_-GFP-Stop(TAA)-(xxx [= the inserted trinucleotide, indicated by lowercase letters])-# clones were created by single or repeated insertions of the four basic trinucleotides and other randomly selected trinucleotides into the 3′-UTR just beyond the stop codon and outside the ORF to amplify the characteristics of each trinucleotide (Additional file 1: Table S3, clones 27–40). In addition, the total MK_6_-GFP expression level was determined (Figure 4A). Several trinucleotide-inserted clones (6×taa, 6×ctc, 1×gag, 3×gag, 6×cac, 6×gaa, 6×ggg, 6×ttt, and 6×cca) showed total MK_6_-GFP expression levels similar to that of the undeleted control clone, MK(AAA)_6_-GFP-LE-6H-Stop(TGA) (Figure 4A, lanes 3–6, 8, 9, and 12–14). However, five trinucleotide-inserted clones (6×gag, 6×aaa, 6×aag, 6×tga, and 6×aga) showed expression levels as low or lower than that of the deleted control clone, MK(AAA)_6_-GFP-Stop(TAA)-# (Figure 4A, lanes 7, 10, 11, 15, and 16).

These results show that the clones with trinucleotide insertions beyond the stop codon in the 3′-UTR had comparable or reduced total protein expression levels compared to those of the undeleted and deleted control clones, respectively. Regarding the homogenous trinucleotide inserts, the 6×ggg and 6×ttt trinucleotide insertions increased total protein expression, whereas 6×aaa did not. The 6×ccc insertion could not be tested because the corresponding complementary oligonucleotide, 6×ggg, could not be synthesized; instead, we synthesized 6×cca, which increased the total protein expression level (Figure 4A, lanes 10, 12, 13, and 14).

Most of the non-homogenous trinucleotide insertions were not consistent with respect to increasing or decreasing protein expression, though protein expression did seem to depend on the number of trinucleotide repeats and nucleotide order. Clones with the trinucleotide inserts of 1*×*gag and 3*×*gag showed increased total MK_6_-GFP expression levels (Figure 4A, lanes 5 and 6), while the clone with the longer insert, 6×gag, showed decreased total MK_6_-GFP expression (Figure 4A, lane 7). Thus, it seems that the length of the trinucleotide insert is important for regulating total protein expression.

Regarding the order of nucleotides, we compared the characteristics of the inserted trinucleotide 6*×*gaa, which behaved as an inducer of protein expression, with those of 6*×*aga and 6*×*aag, which behaved as reducers (Figure 4A, lanes 9, 11, and 16). The three trinucleotide repeated sequences were placed after the TAA stop codon (i.e., TAA-[gaa]_*n* = 6_, TAA-a-[gaa]_*n* = 5_-ga, and TAA-aa-[gaa]_*n* = 5_-g, respectively), and the only difference between the three sequences was the number of “a” nucleotides (none, one, or two, respectively) between TAA and (gaa). These small differences were apparently sufficient to distinguish between inducers and reducers of total protein expression. Further, the mechanisms enacted by 6*×*aga and 6*×*aag for the reduction of protein expression differed in terms of the quantity of mRNA produced (see below). Thus, it appears that there is a precise, discriminating mechanism for the detection of subtle differences in ribonucleotide order beyond the stop codon in the 3′-UTR, and the corresponding ribonucleotide sequence structure influences total protein expression by way of translational and/or transcriptional regulation.

Next, we analyzed mRNA production by semi-quantitative RT-PCR to determine its relationship with total protein expression over time in the trinucleotide-inserted MK(AAA)_6_-GFP-TAA-(xxx)-# clones (Figure 4B). As determined by RT-PCR, some of the trinucleotide-inserted clones (6×taa, 6×ctc, 1×gag, 3×gag, 6×cac, 6×gaa, 6×ggg, 6×ttt, and 6×cca) demonstrated a correlation between high mRNA levels and high total MK_6_-GFP expression levels (Figure 4B, lanes 3–6, 8, 9, and 12–14). Therefore, translation of these trinucleotide-inserted clones that expressed high total MK_6_-GFP levels is dependent upon changes in mRNA tertiary structure.

The 6×aaa, 6×tga, and 6×aga trinucleotide-inserted clones of MK(AAA)_6_-GFP-TAA-(xxx)-# showed relatively high mRNA levels (Figure 4B, lanes 10, 15, and 16), despite very low levels of total protein expression, which were much lower or similar to that of the deleted, parental control clone. Thus, it seems that the low efficiency of translation in these trinucleotide-inserted clones may have been caused by an unchanged mRNA tertiary structure or by a newly formed mRNA tertiary structure. In the case of the 6×gag and 6×aag trinucleotide-inserted clones with low total MK_6_-GFP expression, the mRNA levels according to RT-PCR analysis were also very low or invisible (Figure 4B, lanes 7 and 11), indicating that protein expression was regulated by a transcriptional reducer rather than by a translational reducer and would likely be directly controlled by the quantity of transcript.

In summary, we are the first to demonstrate how changes in the structure and quantity of mRNA, using random screenable trinucleotide insertions beyond the stop codon in the 3′-UTR, function as translational inducers or reducers or transcriptional reducers without changing the ORF sequence. Subtle differences in nucleotide order in trinucleotide insertions beyond the stop codon in the 3′-UTR, outside of the ORF, could introduce more complicated changes in mRNA structure or sequence than expected to affect translational and transcriptional regulation. Furthermore, it seems likely that the location and function of the inserted trinucleotides 6×gag and 6×aag (Figure 4B, lanes 7 and 11) were closely related to Rho-dependent transcriptional termination at the Rho utilization site (*rut*) [8]; however, further study is required to confirm and characterize this mechanism.

## Conclusions

We showed that the hydrophilic C-terminus of GFP could increase Tat pathway specificity for soluble protein expression in expression-induced clones. However, in an expression-reduced clone, we demonstrated that the longer 5′ poly(A) coding sequence had a strong relationship with the 3′ reconstructed nucleotide sequence in the formation of a new mRNA tertiary structure, which resulted in reduced total protein expression. To overcome the low protein expression level by further changing the 3′ reconstructed nucleotide sequence, we showed that total protein expression was affected by a single nucleotide change, as determined by synonymous codon substitutions in the C-terminal 5′ codons and stop codon within the ORF, and that the insertion of trinucleotide sequences, without changing the ORF sequence, beyond the stop codon in the 3′-UTR could control translational or transcriptional regulation. Based on our results, both types of changes in the 3′ region nucleotide sequence are evidently involved in the formation of the critical, specific mRNA structure or in the termination of mRNA transcription, which can influence translational and/or transcriptional regulation, resulting in higher or lower total protein expression levels.

Changes in the nucleotide sequence of the 3′ region showed a clear relationship with the resultant protein expression levels. In the intermediate stage of mRNA processing for low total protein expression levels, there exists a complex mechanism dependent on the tertiary structure that forms between the 5′ and 3′ regions. This mRNA processing mechanism is evidently not easily explained by the established theory of the folding energy of the mRNA secondary structure. However, we clearly demonstrated the existence of an mRNA tertiary structure by changing the nucleotide sequence in the 3′ region and 3′-UTR, which is critical for mRNA translation *in vivo*. To understand this complicated mRNA tertiary structure, future studies should focus on developing a convenient technique for measuring mRNA structures effectively *in vivo* or *in vitro* as well as a new prediction algorithm for evaluating these structures.

Overall, in this study we presented a very easy and useful technique for controlling protein expression levels by manipulating the 3′ region nucleotide sequence within or outside of the ORF. Our results provide important clues to understanding the organization of translational and transcriptional regulation by the 3′ region ribonucleotide sequence, which will be helpful for producing recombinant proteins efficiently as well as for potentially controlling specific genes to increase or decrease expression levels in the body through the engineering of patient-specific cell lines or a pathogenic virus directly, without changing anything within the ORF; such applications have therapeutic implications. We also demonstrated how a gene can be silenced by the insertion of trinucleotides beyond the stop codon in the 3′-UTR, as a self-sequence control regulatory mechanism without pairing an miRNA to the 3′-UTR [9]. These novel screening methods for probing translational and transcriptional control mechanisms of gene regulation may be applicable to all living creatures.

## Methods

### Bacterial strains and plasmids

*Escherichia coli* strains XL-1 blue (Stratagene) and TOP10 (Invitrogen) were used for cloning; BL21(DE3) (Novagen) was used for direct expression of the fusion or unfusion protein. The TA cloning vector (Promega) was used for cloning; pET-22b(+) (Novagen) was used for protein expression.

### Reagents and molecular techniques

Restriction endonucleases from Roche were used. All other chemicals were of analytical grade. All molecular techniques were conducted as described in [10]. Nucleotide sequencing, using the dideoxy chain-termination method [11], was performed using a Sequenase 2.0 kit (United States Biochemical).

### Computational analysis of pI and hydrophilicity, folding energy, and CAI

pI values and hydrophilicity and hydropathy profiles (Hopp & Woods scale) were analyzed using the computer program DNASIS™ (Hitachi, Japan, 1997). Hydrophobicity (hy + or –) (Hopp & Woods scale with window size: 6 and threshold line: 0.00) was calculated using DNASIS™ (Hitachi, Japan, 1997). On the Hopp & Woods scale, hydrophilic regions are given a positive value, and hydrophobic regions a negative one. We calculated RNA secondary structure folding using the RNAfold WebServer [4,5]. The codon adaptation index was calculated using the CAI Calculator 2 [7].

### Construction of GFP clones

To construct GFP expression vectors carrying the native hydrophilic C-terminal sequence (MDELYK, 6 aa, hy +0.35) with a native stop codon (TAA), we removed the LeuGlu (*Xho*I recognition site, CTCGAG)-6×His(His tag, hy −0.28, 6×CAC) peptide from the C-terminus of the GFP fusion protein derived from pET-22b(+)(*N-terminal-gfp-LeuGlu-6*×*His*) (Additional file 1: Table S2). To obtain various K_6_ codon sequences of the MK_6_ N-terminal, we designed various orders of the 5×AAA and 1×AAG codons in the K_6_ sequence for the forward primers (Additional file 1: Table S1). We then designed reverse primers that contained the complementary C-terminal, the complementary C-terminal 5′-codon and stop codon substituted with a synonymous codon, or the complementary trinucleotide inserted singly or repeatedly beyond the stop codon and linked to the complementary *Xho*I cleavage site (CTCGAG) (Additional file 1: Table S2). We amplified the *gfp* region of pEGFP-N2 vector (Clontech) using forward primers containing the *Nde*I cleavage site (CAT) (Additional file 1: Table S1) and the above reverse primers containing the *Xho*I restriction site (Additional file 1: Table S2). The amplified DNA fragment was cloned into a TA cloning vector, the entire *Nde*I-*Xho*I fragment of which was then subcloned into pET-22b(+) by replacing the *pel* signal sequence and polylinker as described previously [12]. Here, we indicate the coding region of the *Xho*I restriction site in (CTCGAG)-6×His(CAC) tag as LeuGlu-6×His(LE-6H) and the non-coding region of the same sequence beyond the stop codon as *XhoI-6*×*His* (referred to as “#” below). The resulting construct, pET-22b(+)(*N-terminal-gfp-stop-#*), and derivative clones containing the synonymous codon-substituted C-terminus and stop codon or trinucleotide inserted beyond the stop codon were obtained (Additional file 1: Table S3).

### GFP expression

*Escherichia coli* BL21 (DE3) cells were transformed with the plasmid constructs listed in Additional file 1: Table S3, and the transformants were cultured in LB medium overnight at 30°C in the presence of 100 μg/mL ampicillin. The culture was then diluted 1:100 in LB medium and grown until it reached an OD_600_ of 0.3. Next, IPTG was added to a final concentration of 1 mM and the culture was grown for another 3 h to allow expression of the recombinant protein. An aliquot was then removed from each culture and centrifuged. The wet weight of the cell pellet was measured and resuspended in Tris buffer (50 mM Tris, pH 8.0). The cells were then disrupted by sonication, in which 15 pulses at 30% power output were applied in 2-s cycles to release total proteins, and the supernatant was obtained as the soluble fraction by centrifugation (16,000 rpm, 30 min, 4°C). Approximately 50 μg of total protein and a counter volume of the soluble fraction were used to measure the fluorescence with a Perkin Elmer Victor3 Multilabel Plate Reader.

### Semi-quantitative RT-PCR analysis of *gfp* mRNA expression

Trizol (Invitrogen, Burlington, ON) was used to extract total RNA from the bacteria according to the manufacturer’s protocol. The quality of the RNA was assessed with ethidium bromide staining and formaldehyde-containing agarose gel electrophoresis. The RNA (1 μg) was then reverse-transcribed into cDNA using random hexamer primers with a Transcriptor First Strand cDNA Synthesis Kit (Roche Diagnostics, Mannheim, Germany).

*gfp* mRNA expression following induction by IPTG for 1 h was evaluated and compared by semi-quantitative RT-PCR. Briefly, the sense primer MK_6_-GFP (F: Additional file 1: Table S1, nos. 3–10) and a universal antisense primer specific for the nucleotide region of # (*XhoI-6×His*) (R: 5′-GTGGTGGTGGTGGTGGTGCTCGAG) were used. The *E. coli* 16S rRNA gene [13] was used as an internal control (RT-F: 5′-CTC CTA CGG GAG GCA GCA G-3′, positions 339-357; RT-R: 5′-CCA GGG TAT CTA ATC CTG-3′, positions 785–768). All PCRs were performed using 5 μL of dNTP mix (2.5 mM each), 0.1 μL of Taq DNA polymerase (TaKaRa, Shiga, Japan), 5 μL of Taq 10× buffer, 1 μL of each primer (10 μM), and 1 μL of cDNA in a final volume of 50 μL. The amplification conditions were: 5 min at 94°C followed by 30 cycles of 30 s at 94°C, 30 s at 55°C, and 1 min at 72°C, with a final extension step of 7 min at 72°C. The products were separated on a 1.5% agarose gel, stained with ethidium bromide, and the bands were photographed.

## Competing interests

The authors declare that they have no competing interests.

## Authors’ contributions

SJL conceived the project, analyzed the data, and wrote the paper. EHP performed the expression trials. All authors have read and approved the final manuscript.

## Supplementary Material

Additional file 1 **Supplementary GFP expression.** Supplementary text describes expression trials using the pET22b(+) plasmid. Figure S1 shows the influence of the substituted 3′ region nucleotide positions and their effects on total protein expression. **Table S1** describes the N-terminals of the GFP clones and the corresponding forward primers used to construct the GFP clone derivatives. **Table S2** describes the various reverse primers used to generate the native hydrophilic C-terminals used to construct the GFP clone derivatives. **Table S3** describes the details of the constructed GFP clones and their locations. Click here for file
