# Supplementary material for: Control of total GFP expression by alterations to the 3′ region nucleotide sequence
Source: Microb Cell Fact. 2013 Jul 8;12:68. doi: 10.1186/1475-2859-12-68 (PMC3726318; doi:10.1186/1475-2859-12-68)
Supplement: Additional file 1 — Supplementary GFP expression. Supplementary text describes expression trials using the pET22b(+) plasmid. Figure S1 shows the influence of the substituted 3′ region nucleotide positions and their effects on total protein expression. Table S1 describes the N-terminals of the GFP clones and the corresponding forward primers used to construct the GFP clone derivatives. Table S2 describes the various reverse primers used to generate the native hydrophilic C-terminals used to construct the GFP clone derivatives. Table S3 describes the details of the constructed GFP clones and their locations. [file 1475-2859-12-68-S1.docx]

**Additional File 1: Supplementary GFP expression.**

**
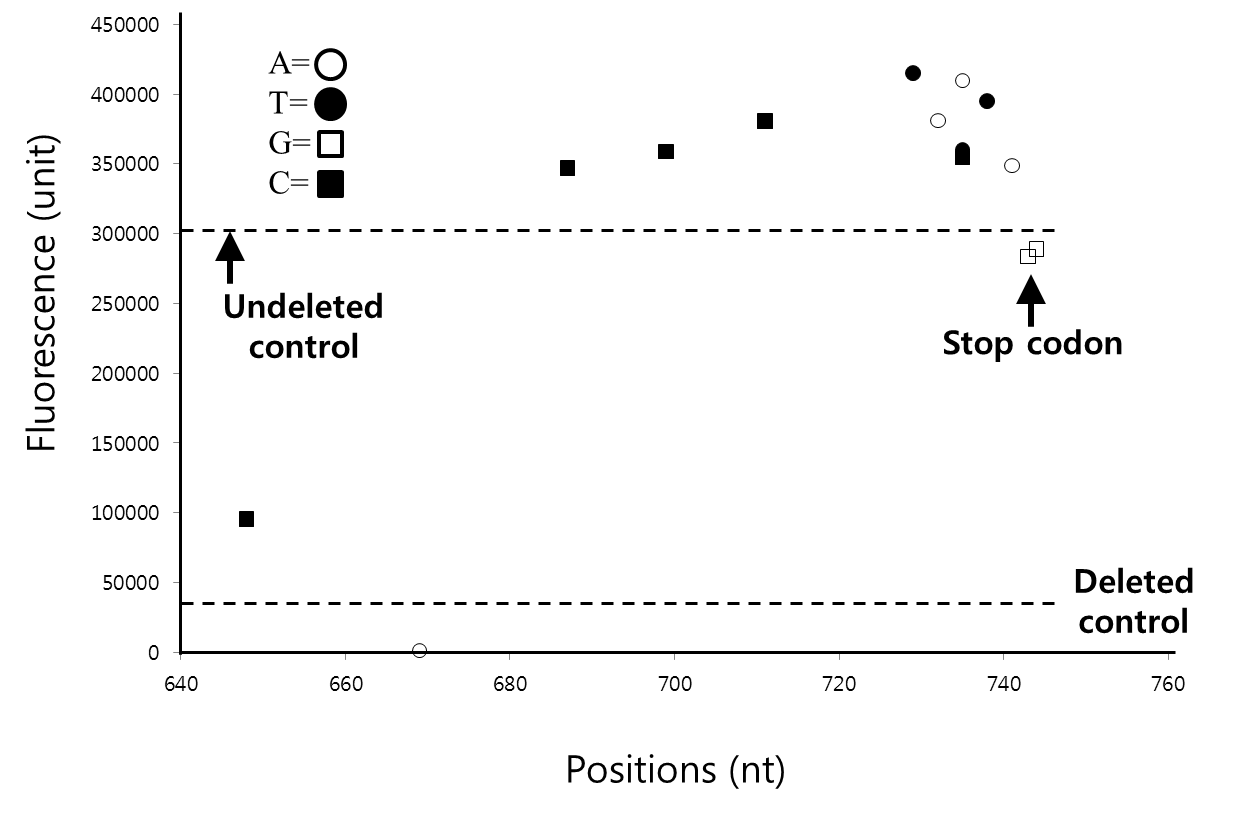
**

**Figure S1.** Influence of the substituted 3’ region nucleotide positions and composition in the various MK_6_-GFP-Stop-# clones on total MK_6_-GFP expression.

We plotted the total MK_6_-GFP expression levels from the MK(AAA)_6_-GFP-Stop-# clones substituted with a synonymous codon at the C-terminal 5’-codons and stop codon at the 3’ region (nt positions 648 to 744), which corresponded to the lanes 1-16 of Figure 3. At the codon positions, the fluorescence levels are represented by symbols for the corresponding G, C, A, or T nucleotides in the second and third base.

**Supplementary Table S1**. N-terminals of the GFP clones and the forward primers used to construct the corresponding GFP clones in pET-22b(+), as shown in Supplementary Table S3.

| No. | N-terminals of the GFP clones | Forward primers for the GFP clones |
| --- | --- | --- |
| 1* | GFP-LeuGlu(LE)-6×His(6H)-Stop(TGA) | CAT**ATGGTGAGCAAGGGCGAGGAG** |
| 2* | ME(GAA)_6_-GFP-LE-6H-Stop(TGA) | CATATGGAAGAAGAAGAAGAAGAA **ATGGTGAGCAAGGGCGAGGAG** |
| 3* | MK(AAA)_6_-GFP-LE-6H-Stop(TGA) | CATATGAAAAAAAAAAAAAAAAAA **ATGGTGAGCAAGGGCGAGGAG** |
| 4 | MK(AAA)_6_-GFP-Stop(TAA)-# | CATATGAAAAAAAAAAAAAAAAAA **ATGGTGAGCAAGGGCGAGGAG** |
| 5 | MK(AAG) _1_(AAA)_5_-GFP-Stop(TAA)-# | CATATGAA**G**AAAAAAAAAAAAAAA **ATGGTGAGCAAGGGCGAGGAG** |
| 6 | MK(AAA)_1_(AAG)_1_(AAA)_4_-GFP-Stop(TAA)-# | CATATGAAAAA**G**AAAAAAAAAAAA **ATGGTGAGCAAGGGCGAGGAG** |
| 7 | MK(AAA)_2_(AAG)_1_(AAA)_3_-GFP-Stop(TAA)-# | CATATGAAAAAAAA**G**AAAAAAAAA **ATGGTGAGCAAGGGCGAGGAG** |
| 8 | MK(AAA)_3_(AAG)_1_(AAA)_2_-GFP-Stop(TAA)-# | CATATGAAAAAAAAAAA**G**AAAAAA **ATGGTGAGCAAGGGCGAGGAG** |
| 9 | MK(AAA)_4_(AAG)_1_(AAA)_1_-GFP-Stop(TAA)-# | CATATGAAAAAAAAAAAAAA**G**AAA **ATGGTGAGCAAGGGCGAGGAG** |
| 10 | MK(AAA)_5_(AAG)_1_-GFP-Stop(TAA)-# | CATATGAAAAAAAAAAAAAAAAA**G** **ATGGTGAGCAAGGGCGAGGAG** |

*The clones and forward primers used to construct the gene encoding the corresponding recombinant GFP fusion proteins were described previously [1].

The C-terminal fusion peptide is presented as LE (LeuGlu, *Xho*I restriction site, CTCGAG)-6H (6×His, His tag, 6×CAC) derived from the expression vector pET22b(+).

# indicates the non-coding nucleotide sequence of the 6×His-tagged *Xho*I restriction site, *XhoI-6×His*.

All forward primers begin with CAT to preserve the *Nde*I site.

Codons presented in capital letters indicate the polynucleotides in the short hydrophilic N-terminal polypeptides.

Codons presented in bold capital letters indicate the polynucleotides at the N-terminus of GFP in the pEGFP‑N2 vector (Clontech).

**Supplementary Table S2**. Reverse primers used to generate the native hydrophilic C-terminus (MDELYK; 6 aa; hy, +0.35) with substituted C-terminal 5′ codons and stop codons or trinucleotide insertions behind the stop codon to construct the corresponding GFP clones using pET-22b(+), as shown in Supplementary Table S3.

| No. | Patterns of the C-terminus, the C-terminal 5′ codons and stop codon substituted with a synonymous codon, and of the trinucleotide inserted behind the stop codon in the GFP clones (aa and nt) | Reverse primers for the GFP clones |
| --- | --- | --- |
| 1 | C-terminal (-MDELYK)  -Stop(TAA) | **CTCGAG**TTACTTGTACAGCTCGTCCATGCC |
| 2 | C-terminal -1 K  (AAG→AAA)-TAA | **CTCGAG**TTA**TTT**GTACAGCTCGTCCATGCCGAGAGTGATCCC |
| 3 | C-terminal -2 Y  (TAC→TAT)-TAA | **CTCGAG**TTACTT**ATA**CAGCTCGTCCATGCCGAGAGTGATCCC |
| 4 | C-terminal -3 L  (CTG→CTA)-TAA | **CTCGAG**TTACTTGTA**TAG**CTCGTCCATGCCGAGAGTGATCCC |
| 5 | C-terminal -3 L  (CTG→CTT)-TAA | **CTCGAG**TTACTTGTA**AAG**CTCGTCCATGCCGAGAGTGATCCC |
| 6 | C-terminal -3 L  (CTG→CTC)-TAA | **CTCGAG**TTACTTGTA**GAG**CTCGTCCATGCCGAGAGTGATCCC |
| 7 | C-terminal -4 E  (GAG→GAA)-TAA | **CTCGAG**TTACTTGTACAG**TTC**GTCCATGCCGAGAGTGATCCC |
| 8 | C-terminal -5 D  (GAC→GAT)-TAA | **CTCGAG**TTACTTGTACAGCTC**ATC**CATGCCGAGAGTGATCCC |
| 9 | C-terminal -11 G  (GGG→GGC)-TAA | **CTCGAG**TTACTTGTACAGCTCGTCCATGCCGAGAGTGAT**GCC**GGCGGCGGTCACGAACTCCAG |
| 10 | C-terminal -15 V  (GTG→GTC)-TAA | **CTCGAG**TTACTTGTACAGCTCGTCCATGCCGAGAGTGATCCCGGCGGCGGT**GAC**GAACTCCAGCAGGACCATGTG |
| 11 | C-terminal -19 L  (CTG→CTC)-TAA | **CTCGAG**TTACTTGTACAGCTCGTCCATGCCGAGAGTGATCCCGGCGGCGGTCACGAACTCCAG**GAG**GACCATGTGATCGCGCTTCTC |
| 12 | C-terminal -25 K  (AAG→AAA)-TAA | **CTCGAG**TTACTTGTACAGCTCGTCCATGCCGAGAGTGATCCCGGCGGCGGTCACGAACTCCAGCAGGACCATGTGATCGCG**TTT**CTCGTTGGGGTCTTTGCTCAG |
| 13 | C-terminal -32 L  (CTG→CTC)-TAA | **CTCGAG**TTACTTGTACAGCTCGTCCATGCCGAGAGTGATCCCGGCGGCGGTCACGAACTCCAGCAGGACCATGTGATCGCGCTTCTCGTTGGGGTCTTTGCT**GAG**GGCGGACTGGGTGCTCAGGTA |
| 14 | C-terminal-Stop(TAG) | **CTCGAG**CTACTTGTACAGCTCGTCCATGCC |
| 15 | C-terminal-Stop(TGA) | **CTCGAG**TCACTTGTACAGCTCGTCCATGCC |
| 16 | C-terminal-Stop(TAA)-6× taa- | **CTCGAG**ttattattattattattaTTACTTGTACAGCTCGTCCATGCC |
| 17 | C-terminal-TAA-6× ctc- | **CTCGAG**gaggaggaggaggaggagTTACTTGTACAGCTCGTCCATGCC |
| 18 | C-terminal-TAA-1× gag- | **CTCGAG**ctcTTACTTGTACAGCTCGTCCATGCC |
| 19 | C-terminal-TAA-3× gag- | **CTCGAG**ctcctcctcTTACTTGTACAGCTCGTCCATGCC |
| 20 | C-terminal-TAA-6× gag- | **CTCGAG**ctcctcctcctcctcctcTTACTTGTACAGCTCGTCCATGCC |
| 21 | C-terminal-TAA-6× cac- | **CTCGAG**gtggtggtggtggtggtgTTACTTGTACAGCTCGTCCATGCC |
| 22 | C-terminal-TAA-6× gaa- | **CTCGAG**ttcttcttcttcttcttcTTACTTGTACAGCTCGTCCATGCC |
| 23 | C-terminal-TAA-6× aaa- | **CTCGAG**ttttttttttttttttttTTACTTGTACAGCTCGTCCATGCC |
| 24 | C-terminal-TAA-6× aag- | **CTCGAG**cttcttcttcttcttcttTTACTTGTACAGCTCGTCCATGCC |
| 25 | C-terminal-TAA-6× ggg- | **CTCGAG**ccccccccccccccccccTTACTTGTACAGCTCGTCCATGCC |
| 26 | C-terminal-TAA-6× ttt- | **CTCGAG**aaaaaaaaaaaaaaaaaaTTACTTGTACAGCTCGTCCATGCC |
| 27 | C-terminal-TAA-6× cca- | **CTCGAG**tggtggtggtggtggtggTTACTTGTACAGCTCGTCCATGCC |
| 28 | C-terminal-TAA-6× tga- | **CTCGAG**tcatcatcatcatcatcaTTACTTGTACAGCTCGTCCATGCC |
| 29 | C-terminal-TAA-6× aga- | **CTCGAG**tcttcttcttcttcttctTTACTTGTACAGCTCGTCCATGCC |

All reverse primers begin with **CTCGAG** to preserve the *Xho*I site.

All reverse primers comprised the complementary sequence of the C-terminus, the complementary C-terminal 5′ codons and stop codon substituted with a synonymous codon, the complementary stop codon, the complementary sequence of the trinucleotide inserted beyond the stop codon of *gfp*, and the complementary non-coding sequence of the *XhoI-6×His* (#), derived from pET-22b(+).

TTA, CTA, and TCA indicate the complementary stop codons of TAA, TAG, and TGA, respectively.

Bold capital letters in the complementary trinucleotides at the C-terminus of GFP indicate the substituted synonymous codon.

Lowercase trinucleotides indicate the complementary trinucleotide sequence inserted singly or repeatedly beyond the stop codon.

Capital letters indicate the complementary polynucleotides at the C-terminus of GFP.

**Supplementary Table S3**. The specified amino acid or nucleotide sequences representing the various N-termini, the C-termini, the substituted C-terminal 5′ codons and stop codon replaced with a synonymous codon, the stop codon, and the trinucleotide inserted beyond the stop codon in the 3′ UTR of the GFP clones. The corresponding GFP clones were constructed as described in the Materials and Methods, and the locations of the GFP clones are shown in the corresponding Figure.

| No. | GFP clones constructed in the pET-22b(+) | Patterns of the C-terminals and the stop codons or of the inserted trinucleotides (aa and nt) | C-terminal  (6 aa) | Figure |
| --- | --- | --- | --- | --- |
| 1* | **GFP-LeuGlu(LE, *Xho*I, CTCGAG)-6× His(6H, CAC)-Stop(TGA)** (control) | LE-6H-Stop(TGA from pET22b+) | HHHHHH  (H_6_) | 1 |
| 2 | **GFP(C-terminal: MDELYK)-Stop(TAA)**-# | C-terminal-TAA(TAA from *gfp*) | MDELYK | 1 |
| 3* | **ME_6_**-**GFP- LE-6H-Stop(TGA)** (control) | LE-6H-TGA | HHHHHH(H_6_) | 1 |
| 4 | **ME_6_-GFP-Stop(TAA)**-# | C-terminal-TAA | MDELYK | 1 |
| 5* | **MK(AAA)_6_-GFP- LE-6H-Stop(TGA)** (control) | LE-6H-TGA | HHHHHH  (H_6_) | 1,2,3,4 |
| 6 | **MK(AAA)_6_-GFP(C-terminal: MDELYK)-Stop(TAA)**-# | C-terminal-TAA | MDELYK | 1,2,3,4 |
| 7 | **MK(AAG)_1_(AAA)_5_-GFP-Stop(TAA)**-# | C-terminal-TAA | MDELYK | 2 |
| 8 | **MK(AAA)_1_(AAG)_1_(AAA)_4_-GFP-Stop(TAA)**-# | C-terminal-TAA | MDELYK | 2 |
| 9 | **MK(AAA)_2_(AAG)_1_(AAA)_3_-GFP-Stop(TAA)**-# | C-terminal-TAA | MDELYK | 2 |
| 10 | **MK(AAA)_3_(AAG)_1_(AAA)_2_-GFP-Stop(TAA)**-# | C-terminal-TAA | MDELYK | 2 |
| 11 | **MK(AAA)_4_(AAG)_1_(AAA)_2_-GFP-Stop(TAA)**-# | C-terminal-TAA | MDELYK | 2 |
| 12 | **MK(AAA)_5_(AAG)_1_-GFP-Stop(TAA)**-# | C-terminal-TAA | MDELYK | 2 |
| 13 | **MK_6_-GFP[C-terminal -1 K(AAG→AAA)] -Stop(TAA)**-# | C-terminal -1 K(AAG→AAA)-TAA | MDELYK | 3 |
| 14 | **MK_6_-GFP[C-terminal -2 Y(TAC→TAT)] -Stop(TAA)**-# | C-terminal -2 Y(TAC→TAT)-TAA | MDELYK | 3 |
| 15 | **MK_6_-GFP[C-terminal -3 L(CTG→CTA)]-Stop(TAA)**-# | C-terminal -3 L(CTG→CTA)-TAA | MDELYK | 3 |
| 16 | **MK_6_-GFP[C-terminal -3 L(CTG→CTT)]-Stop(TAA)**-# | C-terminal-3 L(CTG→CTT)-TAA | MDELYK | 3 |
| 17 | **MK_6_-GFP[C-terminal -3 L(CTG→CTC)]-Stop(TAA)**-# | C-terminal-3 L(CTG→CTC)-TAA | MDELYK | 3 |
| 18 | **MK_6_-GFP[C-terminal -4 E(GAG→GAA)]-Stop(TAA)**-# | C-terminal-4 E(GAG→GAA)-TAA | MDELYK | 3 |
| 19 | **MK_6_-GFP[C-terminal -5 D(GAC→GAT)]-Stop(TAA)**-# | C-terminal -5 D(GAC→GAT)-TAA | MDELYK | 3 |
| 20 | **MK_6_-GFP[C-terminal -11 G(GGG→GGC)]-Stop(TAA)**-*#* | C-terminal -11 G(GGG→GGC)-TAA | MDELYK | 3 |
| 21 | **MK_6_-GFP[C-terminal -15 V(GTG→GTC)]-Stop(TAA)**-*#* | C-terminal -15 V(GTG→GTC)-TAA | MDELYK | 3 |
| 22 | **MK_6_-GFP[C-terminal -19 L(CTG→CTC)]-Stop(TAA)**-*#* | C-terminal -19 L(CTG→CTC)-TAA | MDELYK | 3 |
| 23 | **MK_6_-GFP[C-terminal -25 K(AAG→AAA)]**- **Stop(TAA)-*#*** | C-terminal -25 K(AAG→AAA)-TAA | MDELYK | 3 |
| 24 | **MK_6_-GFP[C-terminal -32 L(CTG→CTC)]**- **Stop(TAA)** -*#* | C-terminal -32 L(CTG→CTC)-TAA | MDELYK | 3 |
| 25 | **MK_6_-GFP-Stop(TAG)**-*#* | C-terminal-TAG | MDELYK | 3 |
| 26 | **MK_6_-GFP-Stop(TGA)** -*#* | C-terminal-TGA | MDELYK | 3 |
| 27 | **MK_6_-GFP-Stop(TAA)**-6× taa | C-terminal-TAA-6× taa | MDELYK | 4 |
| 28 | **MK_6_-GFP-Stop(TAA)**-6× ctc-*#* | C-terminal-TAA-6× ctc | MDELYK | 4 |
| 29 | **MK_6_-GFP-Stop(TAA)**-1× gag-*#* | C-terminal-TAA-1× gag | MDELYK | 4 |
| 30 | **MK_6_-GFP-Stop(TAA)**-3× gag-*#* | C-terminal-TAA-3× gag | MDELYK | 4 |
| 31 | **MK_6_-GFP-Stop(TAA)**-6× gag-*#* | C-terminal-TAA-6× gag | MDELYK | 4 |
| 32 | **MK_6_-GFP-Stop(TAA)**-6× cac-*#* | C-terminal-TAA-6× cac | MDELYK | 4 |
| 33 | **MK_6_-GFP-Stop(TAA)**-6× gaa-*#* | C-terminal-TAA-6× gaa | MDELYK | 4 |
| 34 | **MK_6_-GFP-Stop(TAA)**-6× aaa-*#* | C-terminal-TAA-6× aaa | MDELYK | 4 |
| 35 | **MK_6_-GFP-Stop(TAA)**-6× aag-*#* | C-terminal-TAA-6× aag | MDELYK | 4 |
| 36 | **MK_6_-GFP-Stop(TAA)**-6× ggg-***#*** | C-terminal-TAA-6× ggg | MDELYK | 4 |
| 37 | **MK_6_-GFP-Stop(TAA)**-6× ttt-*#* | C-terminal-TAA-6× ttt | MDELYK | 4 |
| 38 | **MK_6_-GFP-Stop(TAA)**-6× cca-*#* | C-terminal-TAA-6× cca | MDELYK | 4 |
| 39 | **MK_6_-GFP-Stop(TAA)**-6× tga-*#* | C-terminal-TAA-6× tga | MDELYK | 4 |
| 40 | **MK_6_-GFP-Stop(TAA)**-6× aga-*#* | C-terminal-TAA-6× aga | MDELYK | 4 |

*All clones used as controls were constructed previously [1].

Bold letters indicate the ORF (from Met to the stop codon).

‘Codon unspecified MK_6_-’ indicates MK(AAA)_6_-.

Lower case letters indicate the trinucleotide inserted singly or repeatedly just beyond the stop codon.

# indicates the non-coding nucleotide sequence of LeuGlu(*Xho*I restriction site, ctcgag)-6×His(His tag, cac) (*XhoI-6*×*His*), derived from pET-22b(+), located beyond the stop codon and out of frame.
